# Operative Management of Failed Rotator Cuff Repair With Soft Tissue Release

**DOI:** 10.7759/cureus.15970

**Published:** 2021-06-27

**Authors:** Nicholas Bertha, Gary Updegrove, Ghazal Staity, Padmavathi Ponnuru, April Armstrong

**Affiliations:** 1 Orthopaedics, Penn State Health Milton S. Hershey Medical Center, Hershey, USA

**Keywords:** rotator cuff tears, arthroscopic shoulder surgery

## Abstract

Background

We hypothesize that revision surgery that includes soft tissue releases for patients with residual pain and reduced range of motion following rotator cuff repair can provide pain relief and improvement of motion and function.

Methods

Patients were identified via a retrospective chart over a 10-year period who had a history of previous rotator cuff repair and had revision surgery with or without a rotator cuff repair and soft tissue release. Changes in visual analog scores (VAS) pain score on a 10-point scale and shoulder motion including forward flexion and external rotation were evaluated from the preoperative visit to the postoperative visit.

Results

In total, 73 patients underwent procedures to address their symptoms following failed rotator cuff repair. Patients that underwent soft tissue release with revision rotator cuff repair and those who underwent isolated soft tissue release had decreased postoperative VAS pain scores (absolute reduction of 3 and 1.6 points, respectively) and improved postoperative forward flexion (15.3° and 13.6° respectively). Patients that have had one previous surgery had decreased pain (absolute reduction of 3.2 points), increased forward flexion and external rotation (16.2° and 4.9°). Patients that underwent two or more previous surgeries had decreased pain (absolute reduction of 1.8 points) and increased forward flexion (12.7°). Patients who were filing a Worker’s compensation claim also had decreased pain (absolute reduction of 2.2 points) and increased forward flexion (14.9°). Overall, there was a VAS pain scores absolute reduction of 2.6 points or 49.5% when examining the entire patient population.

Conclusion

Operative management by performing soft tissue release with or without concurrent revision rotator cuff repair is successful for both decreasing pain as well as improving motion. This effect was noted both in patients with commercial insurance and worker’s compensation claims. Improvements of pain and motion were more significant in patients who had undergone one prior surgery compared to those who have had multiple prior procedures.

## Introduction

Rotator cuff tears are a common cause of pain and disability, with a prevalence of approximately 20% in the general population and >50% in patients greater than eighty years old [1-4]. While many patients have good outcomes with rotator cuff repair surgery, there is a subset of patients who continue to have pain, stiffness, or decreased function after surgery. Complications from rotator cuff repair include post-operative stiffness (in up to 10% of patients [5]), failure of tendon healing or retear, and post-operative pain. Failure of tendon healing or retear rates are not insignificant, with studies ranging from 21% to 94% of primary repairs and 23% to 71% of revision repairs having a recurrent tear within two years of surgery [6-12].

Risk factors for failure of tendon healing include age, size of the tear, amount of tendon retraction, chronicity of the tear, and fatty degeneration [8,13,14]. There are reports of a subset of patients whom undergo surgery yet continue to have residual pain, decreased range of motion [15], and/or weakness [2,5], many times resulting in lower quality of life [6,16]. Patients with residual symptoms may undergo multiple revision rotator cuff repair operations which may or may not alleviate their symptoms [17]. Patients with residual loss of motion and persistent pain after primary or revision rotator cuff repairs are sometimes counseled that there are few options to manage these challenging symptoms. This is perhaps most noteworthy in patients involved in worker’s compensation claims, who are historically at increased risk for poor outcomes [14].

As the authors gained more experience with treating patients referred for a failed rotator cuff surgery, the importance of recognizing and treating the postsurgical joint contracture that often was associated with these cases became apparent. We hypothesized that patients who underwent a revision surgery following a failed rotator cuff surgery also required soft tissue releases. We also hypothesized that patients with residual pain and decreased range of motion will see improved pain relief and range of motion, both when the previous rotator cuff repair is intact and when a revision repair is indicated. We believe that pain can be improved independent of whether a revision rotator cuff repair is indicated and that soft tissue release alone can be effective to improve pain control. We aim to determine the effects of soft tissue releases as part of the operative management for patients in the setting of failed rotator cuff surgery with residual shoulder pain and stiffness and examined patients who underwent a soft tissue release after failed rotator cuff surgery with or without a revision rotator cuff repair.

## Materials and methods

Patient selection

Patients surgically treated by a single surgeon were identified via a retrospective chart review of cases from Institutional Review Board (IRB) study 00011713 which identified patients from our institution’s enterprise information management system with the Current Procedural Terminology (CPT) code 29825 from 01/01/2010 to 01/01/2019. Inclusion criteria consisted of patients with prior rotator cuff repair who presented with continued pain and stiffness and who subsequently underwent revision surgery to manage their symptoms. The procedures performed were categorized as soft tissue release with revision rotator cuff repair and soft tissue release without revision rotator cuff repair. Patients who were incarcerated, <18 years old at the time of surgery, or pregnant were excluded. Of note, patients with arthritis were not excluded.

Information obtained from chart review included preoperative and postoperative visual analog scale (VAS) pain scores, preoperative and postoperative shoulder flexion, preoperative and postoperative external rotation, number of prior surgeries performed, the surgery performed, and Worker’s compensation status. Utilizing this information, we classified the patients into sub-groups to analyze the effectiveness of surgical intervention based on: Number of prior surgeries, surgery performed (soft tissue release with revision rotator cuff repair vs soft tissue release without revision rotator cuff repair), and insurance status (Worker’s compensation vs commercial insurance).

Analysis

Changes in VAS pain score as well as shoulder motion measurements, including forward flexion and external rotation were calculated. Improvement in pain scores was reported as both percentage of pain relief and absolute VAS reduction from the preoperative visit to the postoperative visit. Paired t-tests and descriptive statistics were performed using Graphpad Prism8 (GraphPad Software, San Diego, CA).

Preoperative clinical assessment

When evaluating patients in clinic with continued pain after rotator cuff repair, it is critical to observe range of motion, particularly forward flexion and external rotation. While it is important to assess active range of motion, perhaps more important in the case of continued pain after repair is the active-assisted or passive range of motion. Patients with persistent pain and reduced range of motion compared to the contralateral shoulder and limited function underwent magnetic resonance imaging (MRI) to evaluate the integrity of the healing repair as well as for other potential pathology such as arthritis at the glenohumeral joint. Patients that had evidence of a recurrent rotator cuff tear >50% thickness on MRI who had persistent symptoms were considered candidates to undergo soft tissue releases with revision rotator cuff repair. Patients that had either <50% thickness tear on MRI or an intact rotator cuff were considered candidates to undergo soft tissue release without revision rotator cuff repair.

Procedure

All surgeries were performed by the senior author. An examination under anesthesia was performed prior to incision to provide a baseline range of motion. Standard anterior, posterior, and lateral arthroscopic portals were utilized. After the initial diagnostic arthroscopy, including evaluation of the rotator cuff tendon, attention was turned to release of the soft tissue structures. This begins while intraarticular with lysis of adhesions, release of the rotator interval and anterior capsule until normal compliance of soft tissues, and release of scarring about the biceps tendon. It is critical to debride the synovium in the rotator interval and work medially toward the coracoid process. The rotator interval adhesions contribute most significantly to the loss of external rotation with the arm in adduction. Often there is synovitis posteriorly in the joint around the posterior labrum which can be debrided. In some cases, the rotator cuff is also scarred to the superior labrum and this can be carefully released to avoid iatrogenic injury to the suprascapular nerve and re-create the normal “gutter” between the labrum and the rotator cuff. Range of motion can be reevaluated intraoperatively with a gentle manipulation to complete the effects of the release. Caution must be taken when debriding anteriorly around the subscapularis to avoid iatrogenic injury of the tendon. When releasing the capsule, particular attention should be taken to the superior and anterior capsule. After completing the anterior release intraarticularly, the subacromial space was entered. A thorough subacromial and subdeltoid decompression including bursectomy was performed with removal of adhesions. Often the normal space between the humeral head/rotator cuff and acromion is obliterated and the surgeon takes time to re-create the normal space between the undersurface of the deltoid and the rotator cuff, particularly if the previous surgery was performed with an open deltoid splitting approach. Finally, the rotator cuff was evaluated. Repair was performed based on surgeon preference depending on the size and repairability of the tear. There was not a standardized protocol for repair, but left to the surgeon’s experience. In general, tears were repaired with suture anchors in a simple fashion. Repairs were performed to avoid significant tension at the time of the repair. Postoperatively, patients that only underwent soft tissue release without a rotator cuff repair started an early range of motion rehab protocol that began on postoperative day one. Patients that underwent a rotator cuff repair had limited weightbearing for six weeks followed the standard rotator cuff repair rehab protocol and began strengthening exercises at three months.

## Results

Patient cohort and demographics

Seventy-three patients were identified and included for analysis. Average age at the time of procedure was 53 years old (range: 25-74 years old). Fifty-five of the 73 patients had a recurrent rotator cuff tear >50% thickness identified on MRI prior to revision surgery and underwent soft tissue release with revision rotator cuff repair. There were no patients in our cohort that had an irreparable tear. The remaining eighteen patients either had an intact or healed rotator cuff or partial thickness tear <50% thickness which was not indicated for repair and underwent soft tissue release without revision rotator cuff repair or debridement. The majority of patients had right-sided repair (n = 43). Average postoperative follow-up time was eight months with a range of three to 29 months. Only one patient had the minimum follow-up of three months and was then lost to follow up for other medical issues. Sixty-nine of the patients included in this study identified as white, two identified as black, and two identified as Hispanic. Forty-six were male and 27 were female (see Table 1). On average, patients had undergone 1.6 previous shoulder surgeries (see Table 2). There was a higher percentage of some degree of arthritis in patients who had ≥2 surgeries (64%) compared to those with one prior surgery (26%) based on recorded data from operative reports (see Table 3).

**Table 1:** Patient demographics. Demographics were obtained by how the patient self-identified. Our patient population predominantly identified as white and male.

Race	Female	Male	Total
Black	1	1	2
Hispanic, Latino, or Spanish descent	1	1	2
White, not Hispanic, Latino, or Spanish origin	24	43	67
White, unspecified	1	1	2
Total	27	46	73

**Table 2:** Number of prior surgeries. Table shows that number of prior surgeries our patient cohort had. Patients most commonly had one prior surgery (62%).

Number of prior surgeries	Number of patients
1	45
2	17
3	6
4	5

**Table 3:** Arthritis grades compared to the number of prior surgeries. Grade of arthritis was determined intraoperatively by the senior author. Most of the patients in our study had mild osteoarthritic changes.

Grade of arthritis	Number of prior surgeries			
Normal	1	2	3	4
1	28	6	3	0
2	2	1	0	0
3	0	1	1	0
4	2	1	1	0
Not recorded	7	1	0	2

There were no patients presenting with recurrent rotator cuff tear who underwent soft tissue release alone without repair. All patients who underwent surgery and were found to have a rotator cuff tear >50% tendon width underwent revision repair in addition to the soft tissue release procedure.

Number of prior surgeries

**Figure 1:**
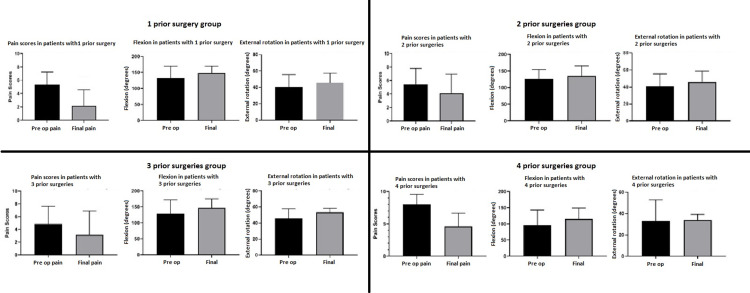
Pain scores and range of motion changes based on number of prior surgeries. Patient preoperative and postoperative VAS pain scores, shoulder flexion in degrees, and external rotation in degrees were recorded and patients were then grouped based on the number of prior surgeries.  In general, there is a trend of decreasing pain and increased range of motion scores throughout all groups, most significant in patients with one prior surgery. ER: external rotation; VAS: visual analog scale.

Patients who had underwent one or two prior shoulder procedures showed a statistically significant postoperative improvement in both pain and motion, while those with three or four prior procedures did not have statistically significant improvement in their pain or motion, though a trend of improvement of both pain and motion was seen. However, when grouped together, patients that underwent ≥2 surgeries had a decrease in pain and an increase in forward flexion (see Table 4 and Figure 1).

**Table 4:** Number of prior shoulder surgeries and effect on clinical outcomes following soft tissue release with or without rotator cuff repair. Patients were categorized based on the number of prior surgeries. Table shows each group’s corresponding change in VAS pain scores (absolute change and percent change), increase in forward flexion in degrees and increase in external rotation in degrees. All values are associated with a 95% confidence interval and P-value. VAS: visual analog scale.

Number of prior shoulder surgeries	N	VAS pain score reduction		Forward flexion increase	External rotation increase
		Absolute	%		
1	45	3.2 (2.4 to 3.9), P<0.0001	60.3% (47.4 to 73.1)	16.2 (7.7 to 24.8), P<0.001	4.9 (0.4 to 9.4), P<0.05
2	17	1.3 (0.3 to 2.4), P=0.01	27.0% (5.9 to 48.0)	8.8 (-7.0 to 24.7), P=0.26	5.1 (-3.6 to 13.8), P=.23
3	6	3.4 (-0.2 to 7.0), P=0.30	43.8% (-34.0 to 121.5)	18.3 (-12.4 to 49.1), P=0.19	7.5 (-4.8 to 19.8), P=0.18
4	5	3.4 (-0.2 to 7.0), P=0.06	40.2% (4.2 to 76.1)	1.0 (-21.6 to 23.6), P=0.20	1.0 (-21.6 to 23.6), P=0.91
≥2	28	1.8 (0.8 to 2.7), p<0.001	32.7% (14.7 to 50.7)	12.7 (1.3 to 24.0), P<0.05	4.9 (-1.2 to 11.0), P=0.11

Procedure performed

**Figure 2:**
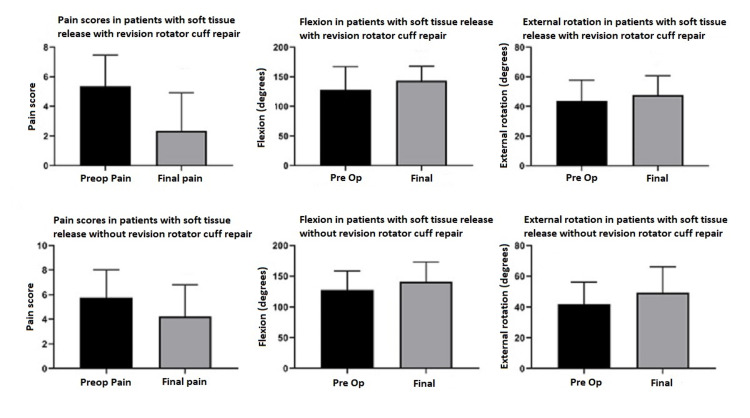
Pain scores and range of motion changes based on the procedure performed. Patient preoperative and postoperative VAS pain scores, shoulder flexion in degrees, and external rotation in degrees were recorded and patients were then grouped based on the procedure performed. In general, there is a trend of decreasing VAS pain scores and increased range of motion scores in both patients that underwent soft tissue release with revision rotator cuff repair and without revision rotator cuff repair. ER: external rotation; VAS: visual analog scale.

Patients had statistically significant post-operative improvements of pain and motion both with and without revision repair of the rotator cuff (see Table 5 and Figure 2).

**Table 5:** Surgical procedure performed and effect on clinical outcomes. Patients were categorized based on the procedure they underwent (soft tissue release without revision rotator cuff repair or soft tissue release with rotator cuff repair). Table shows each group’s corresponding change in VAS pain scores (absolute change and percent change), increase in forward flexion in degrees, and increase in external rotation in degrees. All values are associated with a 95% confidence interval and P-value. VAS: visual analog scale.

Procedure performed	N	VAS pain score reduction		Forward flexion increase	External rotation increase
		Absolute	%		
Soft tissue release with revision rotator cuff repair	55	3.0 (2.3 to 3.7), P<0.0001	56.6% (43.1 to 69.3)	15.3 (7.4 to 23.2), P=0.0003	4.0 (0.2 to 7.9), P=0.04
Soft tissue release without revision rotator cuff repair	18	1.6 (0.7 to 2.4), P=0.002	29.8% (10.2 to 49.3)	13.6 (0.0 to 27.2), P=0.05	7.5 (-1.3 to 16.3), P=0.09

Insurance status

**Figure 3:**
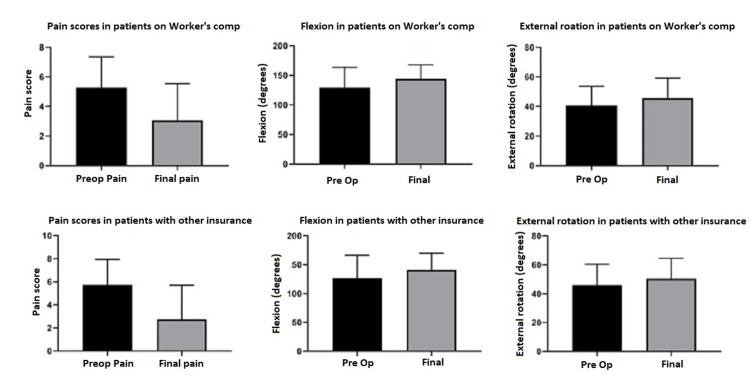
Pain scores and range of motion changes based on insurance status. Patient preoperative and postoperative VAS pain scores, shoulder flexion in degrees, and external rotation in degrees were recorded and patients were then grouped based on insurance status. In general, there is a trend of decreasing pain and increased range of motion scores in both patients with Worker’s compensation claims and those with other insurance. ER: external rotation; VAS: visual analog scale.

Patients had statistically significant post-operative improvements of pain and motion both with Worker’s compensation claims (n = 35) as well as with other insurance payers (n = 38) (see Table 6 and Figure 3).

**Table 6:** Insurance status relationship to clinical outcomes following soft tissue release with or without concurrent rotator cuff repair. Table shows each group’s corresponding change in VAS pain scores (absolute change and percent change), increase in forward flexion in degrees, and increase in external rotation in degrees. All values are associated with a 95% confidence interval and p-value. VAS: visual analog scale.

Insurance status	N	VAS pain score reduction		Forward flexion increase	External rotation increase
		Absolute	%		
Worker’s compensation	35	2.2 (1.4 to 3.0), P<0.0001	43.4% (27.7 to 59.1)	14.9 (5.2 to 24.5), P=0.004	5.3 (-0.1 to 10.7), P=0.05
Other insurance	38	3.0 (2.1 to 3.9), P<0.0001	54.9% (40.0 to 70.1)	14.9 (5.2 to 24.6), P=0.004	4.5 (-0.4 to 9.4), P=0.07

Entire patient population

Overall, there were statistically significant post-operative improvements of pain and motion when examining the entire population.

Patients had a VAS pain score reduction of 2.6 or 49.5% (P < 0.0001). The average improvement of forward flexion was 14.7° (P < 0.0001) and external rotation was 4.8° (P = 0.007).

## Discussion

Performing a soft tissue release was effective in improving pain as a primary surgical intervention when a patient had residual pain and stiffness without a recurrent tear. Soft tissue release was likely an important adjunctive intervention for patients with residual pain and stiffness in conjunction with a revision rotator cuff repair, but it is hard to know how much the soft tissue release or the rotator cuff repair helped in alleviating pain. This effect was most significant in patients who had fewer previous surgeries (most significant with one previous surgery) where results demonstrated both decreased pain scores and improved range of motion. While there was not a statistically significant change in pain reduction and shoulder motion, there was a general trend showing improvement in both pain and range of motion in patients that had underwent three or four previous procedures. A notable finding was performing soft tissue releases with or without concurrent rotator cuff repair lead to decreased pain scores and improved range of motion in patients with Worker’s compensation claims, as well as those with commercial insurance. We would propose that patients who have failed previous rotator cuff surgery often present with a concomitant shoulder contracture and can benefit from soft tissue release procedures alone. When describing the effectiveness of performing soft tissue release with or without RCR, patients demonstrated approximately 50% reduction in pain.

The effectiveness of soft-tissue releases can be attributed to the broad statement that stiff shoulders are painful shoulders. While patients with pain after rotator cuff surgery do not have the same pathophysiology as patients with adhesive capsulitis, they both often present with pain, specifically worsened at the extremes of motion, and limited range of motion. Postoperative stiffness after RCR differs from adhesive capsulitis as the pathology is the result of contracture from surgical scaring, subacromial scaring, or tightness from tendon repair. While adhesive capsulitis often resolves with non-operative management, post-surgical shoulder stiffness is not as likely to resolve with nonoperative management. Once a patient develops a chronic postsurgical rotator interval contracture, it can be very difficult for them to gain this motion back with therapy alone, and perhaps operative soft tissue releases should be considered for these patients.

Areas that were targeted for postoperative shoulder stiffness were the rotator interval, the capsule, the subacromial space, and the subdeltoid space. These areas were debrided to mobilize the surrounding soft tissues and address limitations with external rotation and forward flexion. Our results show that revision rotator cuff repair is only part of providing patients with clinical improvement following previous failed rotator cuff surgery.

Almost half of the patients included in this study were receiving care under a Worker’s compensation claim (n = 35). Prior studies have shown patients with worker’s compensation claims undergoing revision rotator cuff repairs have worse clinical outcomes when compared to patients with commercial insurance [14,17]. Over time this has propagated the conclusion that Worker’s compensation patients were challenging to treat and have increased risk of poor outcomes. This has led many physicians to feel that there are few options available for these patients’ pain and their poor outcomes may have to be expected. However, Worker’s compensation patients in our study whom underwent soft tissue release procedures had a significant improvement in pain and range of motion despite insurance status. We believe that with appropriate treatment, these previously challenging to treat patients could obtain improved clinical outcomes, quality of life, and relieve frustration.

Some other alternatives have been suggested for managing failed rotator cuff repairs. Many methods focus on addressing the rotator cuff tear itself, such as performing tendon transfers; however, most do not address managing pain or range of motion specifically. One method focused on addressing pain and movement is Matsen et al. smooth and move procedure. The focus of this procedure is to smooth the proximal humerus and coracoacromial arch and then working with physical therapy to make gains in range of motion which helps patients stay mobile and limit pain [18,19].

One limitation to this study is that data were obtained retrospectively. Method of measurement of range of motion was not always recorded, and unable to be determined if they were obtained through visual inspection or use of a goniometer. Range of motion angles were obtained by two different observers (the attending surgeon and an advanced practice provider) and no interobserver reliability was assessed at the time of obtaining values. These factors could have led to an underestimated or overstated range of motion change.

Another limitation to this study is the small sample size. We could not sufficiently support statistical significance for patients with two, three, or four previous surgeries. However, there was a trend in the data where most patients had substantial pain reduction and improved range of motion. The few outliers that were present in the small group size had a substantial effect on the results. When examining the entire cohort of patients that had underwent ≥2 surgeries (n = 28), soft tissue release did have statistically and clinically significant improvement in pain and shoulder flexion. This supports that soft tissue release with or without revision rotator cuff repair for residual pain and stiffness could potentially be effective for this population, but would require a larger analysis.

## Conclusions

Performing soft tissue release with or without concurrent revision rotator cuff repair improves pain and motion for patients with residual pain and stiffness after previous rotator cuff repair. It is important to note that those patients with a healed rotator cuff repair also benefitted from surgery, and thus these patients should not be ignored. This was demonstrated for both insured and worker’s compensation patients. Performing soft tissue releases for these patients may be an answer to improve their symptoms. We recommend that patients with persistent pain and decreased range of motion in the setting of previous rotator cuff may benefit from operative soft tissue release. This procedure could be beneficial with or without revision rotator cuff repair and should also be considered in symptomatic patients with healed rotator cuff tendon repairs.
